# Prognostic significance of cytogenetic risk score in patients with secondary acute myeloid leukemia undergoing allogeneic stem cell transplantation from HLA-matched unrelated donors: a study from the ALWP /EBMT

**DOI:** 10.1038/s41409-025-02620-3

**Published:** 2025-05-29

**Authors:** Arnon Nagler, Allain-Thibeault Ferhat, Sarah Kayser, Matthias Eder, Nicolaus Kröger, Matthias Stelljes, Johan Maertens, Régis Peffault de Latour, Igor Wolfgang Blau, Thomas Schroeder, Péter Reményi, Tobias Gedde-Dahl, Gesine Bug, Didier Blaise, Ali Bazarbachi, Jordi Esteve, Mohamad Mohty, Fabio Ciceri

**Affiliations:** 1 Division of Hematology, Sheba Medical Center, Tel Hashomer, Israel; 2 EBMT Paris study office; Department of Haematology, Saint Antoine Hospital; INSERM UMR 938, Sorbonne University, Paris, France; 3 Department of Hematology, Oncology and Cancer Immunology, Charité—Universitätsmedizin Berlin, corporate member of Freie Universität Berlin and Humboldt-Universität zu Berlin, Berlin, Germany; 4 Hannover Medical School, Hannover, Germany; 5 University Hospital Eppendorf, Hamburg, Germany; 6 University of Muenster, Muenster, Germany; 7 University Hospital Gasthuisberg, Leuven, Belgium; 8 Saint-Louis Hospital, BMT Unit, Paris, France; 9 Medizinische Klinik m. S. Hämatologie, Onkologie und Tumorimmunologie, Berlin, Germany; 10 University Hospital Essen, Essen, Germany; 11 Dél-pesti Centrumkórház, Budapest, Hungary; 12 Oslo University Hospital, Rikshospitalet, Oslo, Norway; 13 University Hospital Frankfurt - Goethe University, Frankfurt Main, Germany; 14 Programme de Transplantation & Therapie Cellulaire, Marseille, France; 15 Hematology-Oncology Division, Department of Internal Medicine, American University of Beirut Medical Center, Beirut, Lebanon; 16 Hematology Department, Hospital Clínic, IDIBAPS, University of Barcelona, Barcelona, Spain; 17 Sorbonne University, Department of Haematology, Saint Antoine Hospital; INSERM UMR 938, Paris, France; 18 Hematology & Bone Marrow Transplant, IRCCS San Raffaele Scientific Institute, Milan, Italy

**Keywords:** Acute myeloid leukaemia, Medical research

## Abstract

The cytogenetic risk category retains a pivotal role in the prediction of prognosis in acute myeloid leukemia (AML) patients undergoing hematopoietic stem cell transplantation (HSCT), however, its impact on secondary AML (sAML) is less established. We assessed whether the ELN 2022 cytogenetic risk score predicts outcomes in sAML patients in remission undergoing HSCT from HLA-matched donors performed between 2010 and 2022. Among 1119 patients, 829 had intermediate and 284 had adverse cytogenetics (6 with favorable risk were excluded). Engraftment rates was 72.4% vs. 99.5%. Acute graft-versus-host disease (GVHD) incidence did not differ, but 2-years all grades and extensive chronic GVHD were higher in the intermediate vs. adverse cytogenetics risk groups, hazard ratio (HR) = 0.72; *p* = 0.034 and HR = 0.58; *p* = 0.027, respectively. Two-year non-relapse mortality (NRM) was similar. All other HSCT outcomes were inferior in the adverse risk vs. intermediate-risk patients: The HR for 2-year relapse incidence (RI) was 2.48 (95% CI 1.95–3.15, *p* < 0.001). The HRs for 2-year leukemia-free survival (LFS), overall survival (OS), and GVHD-free/relapse-free survival (GRFS) were 1.62 (95% CI 1.34–1.95, *p* < 0.001), 1.59 (95% CI 1.3–1.93, *p* < 0.001) and 1.38 (95% CI 1.15–1.65, *p* < 0.001), respectively. We conclude that cytogenetic risk score predicts HSCT outcomes in sAML patients.

## Introduction

Secondary acute myeloid leukemia (sAML) comprises a heterogeneous group of diseases evolving from a preexisting hematologic disorder, predominantly myelodysplastic syndrome (MDS) or myeloproliferative disorders (MPD), or as a complication of prior cytotoxic chemotherapy or radiation therapy [1–8]. sAML has been associated with inferior outcomes compared to de novo AML due to factors such as the antecedent hematological disorder, older age, more aggressive biology of the leukemia with adverse cytogenetics and a high-risk mutation profile, lower chemotherapy susceptibility and reduced treatment tolerance, among others [1, 6, 7, 9]. Allogeneic stem hematopoietic cell transplantation (HSCT) remains the only known potentially curative therapy [10–15]. While HSCT is commonly employed in fit de novo AML patients with high-risk cytogenetics to mitigate relapse risk, significant post-transplant relapse rates are persist in this high-risk patient population [6–8, 16]. The cytogenetic risk category retains a pivotal role in predicting prognosis in AML patients owing to its tight association with survival and heightened risk of disease relapse [6–8, 16]. Currently, the prognosis of AML patients is determined by cytogenetic risk score and karyotypic abnormalities. However, it remains unclear whether the cytogenetic risk group, assessed at baseline, retains similar prognostic value in patients with high risk or active disease referred for HSCT [17–19]. We previously studied the impact of baseline cytogenetic risk on various transplantation outcomes in patients with relapsed/refractory (R/R) de novo AML with active disease undergoing HSCT. In multivariate analysis (MVA), the relapse incidence (RI) was significantly higher, and leukemia-free survival (LFS) and overall survival (OS), were significantly lower for patients with adverse-risk cytogenetics compared to those with intermediate-risk cytogenetics [17]. Furthermore, we assessed the prognostic impact of cytogenetics in patients with AML harboring FMS-like tyrosine kinase 3 internal tandem duplication (FLT3-ITD), as it remained unclear whether baseline cytogenetics significantly impacts the post-HSCT outcome in these patients. Our study demonstrated that the cytogenetic risk category retained its prognostic impact in transplanted high-risk FLT3-ITD AML patients. In MVA, LFS and OS were significantly lower and relapse higher in patients with adverse risk cytogenetics, and intermediate and compared with those with favorable risk cytogenetics [18]. Finally, we assessed the prognostic impact of cytogenetics risk in AML patients with positive pre-HSCT measurable residual disease (MRD). Once more, the cytogenetic risk score retained its prognostic impact in transplanted MRD^+^ AML patients [19]. However, no prior study has evaluated the prognostic impact of cytogenetic risk in sAML patients undergoing HSCT. This assessment is of particular clinical importance, as sAML is a high risk leukemia and thus, in contrast to de novo AML, with favorable risk cytogenetics is referred to HSCT in first complete remission. We therefore assessed the prognostic significance of the LeukemiaNet (ELN) 2022 cytogenetic risk score in patients with sAML undergoing HSCT from HLA matched siblings (MSD) or matched unrelated donors (MUD) using the dataset of the Acute Leukemia Working Party (ALWP) of the European Society for Blood and Marrow Transplantation (EBMT).

## Patients and methods

### Study design and data collection

This was a retrospective, multicenter analysis using the dataset of the ALWP of the EBMT. The EBMT is a voluntary working group of more than 600 transplant centers that are required to report all consecutive stem cell transplantations and follow-ups once a year. EBMT minimum essential data forms are submitted to the registry by transplant center personnel following written informed consent from patients in accordance with the centers’ ethical research guidelines. Data accuracy is assured by the individual transplant centers and by quality control measures such as regular internal and external audits. In addition, the study protocol was approved by each site and complied with country-specific regulatory requirements.

Eligibility criteria for this analysis included adult patients ≥18 years of age with sAML post-MDS or MPD in first complete remission (CR1) who underwent a first HSCT from a human leukocyte antigen (HLA) matched sibling donor (MSD) or 10/10 HLA matched unrelated donor MUD 2010 and 2022. Exclusion criteria were HSCT from other donor types (haploidentical or cord blood donor), prior HSCT, ex vivo T cell-depleted hematopoietic cell graft, and disease status beyond CR1 at the time of transplantation. Data collected included recipient and donor characteristics (age, gender, cytomegalovirus (CMV) serostatus, and Karnofsky performance status (KPS)), disease characteristics, year of transplant, type of conditioning regimen, stem cell source, and GVHD prophylaxis regimen. The conditioning regimen was defined as myeloablative (MAC) when containing total body irradiation (TBI) with a dose >6 Gray or a total dose of busulfan (Bu) > 8 mg/kg or >6.4 mg/kg when administered orally or intravenously, respectively. All other regimens were defined as reduced intensity conditioning (RIC) [20]. Grading of acute (a) GVHD was performed using established criteria [21]. Chronic (c) GVHD was classified as limited or extensive according to published criteria [22]. For this study, all necessary data were collected according to the EBMT guidelines, using the EBMT minimum essential data forms. A list of institutions contributing data to this study is provided in the Supplemental Appendix.

### Statistical analysis

The median, range, and interquartile range (IQR) were used to express quantitative variables and frequency and percentage for categorical variables. The study endpoints were OS, LFS, relapse incidence (RI), non-relapsed mortality (NRM), and engraftment, aGVHD, cGVHD, and GVHD-free, relapse-free survival (GRFS). All endpoints were measured from the time of transplantation. Myeloid engraftment was defined as achieving an absolute neutrophil count of ≥0.5 × 10^9^/L at day 30 for three consecutive days. Platelet engraftment was defined as achieving a platelet count of ≥20 × 10^9^/L at day 60 for three consecutive days. OS was defined as time to death from any cause. LFS was defined as survival with no evidence of relapse or progression. NRM was defined as death from any cause without previous relapse or progression. We used modified GRFS criteria. GRFS events were defined as the first event among grade III-IV aGVHD, extensive cGVHD, relapse, or death from any other cause [23]. Patient, disease, and transplant-related characteristics were compared using the Mann–Whitney *U* test for numerical variables, and the chi-squared or Fisher’s exact test for categorical variables. The probabilities of OS, LFS, and GRFS were calculated using the Kaplan–Meier estimate. The RI and NRM were calculated using cumulative incidence functions in a competing risk setting, with death in remission being treated as a competing event for relapse. Early death was considered as a competing event for engraftment. To estimate the cumulative incidence of aGVHD or cGVHD, relapse, and death were considered as competing events. Multivariate analyses were performed using the Cox proportional-hazards regression model [24]. Results were expressed as the hazard ratio (HR) with a 95% confidence interval (95% CI). All *p* values were two-sided with a type 1 error rate fixed at 0.05. Statistical analyses were performed with SPSS 27.0 (SPSS Inc., Chicago, IL, USA) and R 4.3.2 (R Core Team Fifty (2020). R: A language and environment for statistical computing. R Foundation for Statistical Computing, Vienna, Austria. URL https://www.R-project.org/) [25].

## Results

### Patient, disease, and transplant-related characteristics

A total of 1119 patients met the inclusion criteria. The cytogenetic risk scores were as follows: intermediate in 829 patients and adverse in 284. Six patients with favorable cytogenetics were not included in the analysis. The median follow-up was 3.1 years (range, 2.9–3.7) for patients with intermediate and 4.0 years (range, 3–4.1) for those with adverse risk cytogenetics. The median age was 61.9 (range 18.5–74.9) vs. 61.0 (18.6-74.9) years (*p* = 0.20). Males comprised 62% and 55% of the intermediate- and adverse-risk groups, respectively (*p* = 0.043). The median year of the transplant was 2018 and 2017 (range, 2010–2021 in both) (*p* = 0.34). The antecedent hematological disease for all patients was MDS/MPD (*p* = 0.12). Donors were siblings in 34% vs. 38% and unrelated in 66% and 62%, respectively (*p* = 0.22). The graft source was mobilized peripheral blood stem cells (PB) in 94% and 97% of the patients with intermediate and adverse risk cytogenetics, respectively (*p* = 0.084). Performance status, patient and donor CMV seropositivity, and female-to-male combination did not differ between the groups (Table 1). The median time from diagnosis to HSCT was 4.5 (range 0.6–17.7) compared to 4.4 (0.9–16.6) months (*p* = 0.11). Sixty-one percent and 64% of the patients in both groups received RIC (*p* = 0.1), with busulfan/fludarabine (Flu) being the most frequent regimen for both groups (45% vs. 46%) to be followed by treosulfan/Flu (17% in both) (*p* = 058) (Supplementary Table S1). GVHD disease prophylaxis was cyclosporine A (CSA)/mycophenolate mofetil in 35% if the intermediate-risk group and 38% of the adverse-risk group, while CSA/methotrexate was used in 33% vs. 37%, respectively. Anti-thymocyte globulin was administered to 62% vs. 66% of patients, while post-transplant cyclophosphamide was given to 6.3% vs. 5.6%, respectively (*p* = 0.68) (Supplemental Table S2).

**Table 1:** Patient and Transplant Characteristics.

		Cytogenetic AML classification		*p* value
Variable	Overall *N* = 1113	Intermediate *N* = 829	Adverse *N* = 284	
**Age of the Patient at HSCT (years)**				0.20
Median	61.8	61.9	61.0	
Range	18.5, 74.9	18.5, 74.9	18.6, 74.9	
**Sex of the Patient**				0.043
Female	445 (40%)	317 (38%)	128 (45%)	
Male	668 (60%)	512 (62%)	156 (55%)	
**Year of transplantation**				0.34
Median	2018.0	2018.0	2017.0	
Range	2010.0, 2022.0	2010.0, 2022.0	2010.0, 2022.0	
**Median Follow-up**	3.3 (3–3.8)	3.1 (2.9–3.7)	4 (3–4.1)	
**Months between diagnosis and HSCT**				0.11
Median	4.5	4.5	4.4	
Range	0.6, 17.7	0.6, 17.7	0.9, 16.6	
**Previous diagnosis**				0.12
MDS	824 (74%)	601 (72%)	223 (79%)	
MPN	24 (2.2%)	20 (2.4%)	4 (1.4%)	
MDS or MPN	265 (24%)	208 (25%)	57 (20%)	
**Karnofsky score**				0.22
>=90	702 (66%)	530 (67%)	172 (63%)	
<90	357 (34%)	257 (33%)	100 (37%)	
Missing	54	42	12	
**NPM1 Mutation**				<0.001
Negative	286 (74%)	217 (70%)	69 (88%)	
Positive	102 (26%)	93 (30%)	9 (12%)	
Missing	725	519	206	
**FLT3-ITD Mutation**				0.062
Negative	307 (74%)	232 (72%)	75 (82%)	
Positive	108 (26%)	91 (28%)	17 (18%)	
Missing	698	506	192	
**Type of donors**				0.22
MSD	386 (35%)	279 (34%)	107 (38%)	
UD 10/10	727 (65%)	550 (66%)	177 (62%)	
**Sex of the Donor**				0.49
Female	373 (34%)	273 (33%)	100 (35%)	
Male	735 (66%)	552 (67%)	183 (65%)	
Missing	5	4	1	
**Female donor to male patient**				0.21
No	929 (84%)	685 (83%)	244 (86%)	
Yes	183 (16%)	143 (17%)	40 (14%)	
Missing	1	1	0	
**Patient CMV**				0.37
Negative	366 (33%)	279 (34%)	87 (31%)	
Positive	742 (67%)	547 (66%)	195 (69%)	
Missing	5	3	2	
**Donor CMV**				0.66
Negative	516 (47%)	381 (47%)	135 (48%)	
Positive	584 (53%)	438 (53%)	146 (52%)	
Missing	13	10	3	
**Cell source**				0.084
BM	57 (5.1%)	48 (5.8%)	9 (3.2%)	
PB	1056 (95%)	781 (94%)	275 (97%)	
**vRIC or MAC regimen**				0.10
RIC	685 (62%)	503 (61%)	182 (64%)	
MAC-TBI	45 (4.0%)	29 (3.5%)	16 (5.6%)	
MAC-Chemo	383 (34%)	297 (36%)	86 (30%)	
**Main GVHD Prevention**				0.68
ATG	705 (63%)	517 (62%)	188 (66%)	
PTCY	68 (6.1%)	52 (6.3%)	16 (5.6%)	
PTCY + ATG	13 (1.2%)	11 (1.3%)	2 (0.7%)	
Other	327 (29%)	249 (30%)	78 (27%)	

*HSCT* hematopoietic stem cell transplantation, *sAML* secondary acute myeloid leukemia, *MDS* myelodysplastic syndrome, *MPD* myeloproliferative disorders, *CMV* cytomegalovirus, *BM* bone marrow, *PB* mobilized peripheral blood stem cells, *UD* unrelated donor, *MSD* matched sibling donor, *KPS* Karnofsky performance score (KPS), *ATG* T-cell depletion, *PTCY* post transplantation cyclophosphamide, *FLT3-ITD-FMS* like tyrosine kinase 3 internal tandem duplication, *NPM1* nucleophosmin -1, *RIC* reduced intensity conditioning, *MAC* myeloablative conditioning, *TBI* total body irradiation.

### Transplantation outcomes

**Fig. 1:**
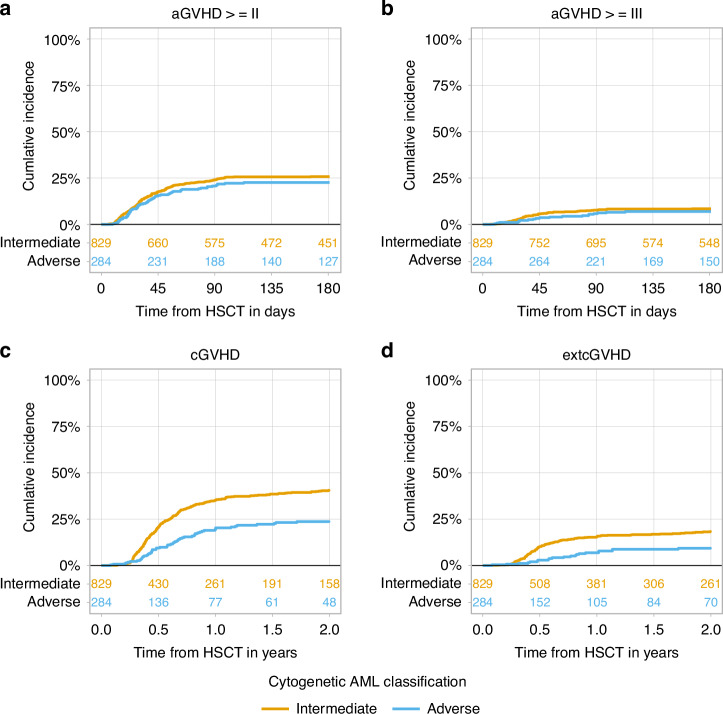
The impact of cytogenetic risk on allogeneic stem cell transplantation outcomes in patients with secondary acute myeloid leukemia. **a** (aGVHD>=II) acute graft versus hose disease grade II-IV; **b** (aGVHD>=III) acute graft versus hose disease grade III-IV; **c** (cGVHD) all grades chronic graft versus host disease; **d** (extcGVHD) extensive chronic graft versus host disease.

The day 30 cumulative incidence of neutrophil engraftment was 96.6% (95% CI 95.1– 97.6%) in the intermediate risk group and 95.3% (95% CI 92–97.3%) in the adverse risk group (Table 2A). The day 60 cumulative incidence of platelet engraftment was 95.3% (95% CI 93.6–96.6%) vs. 92.9% (95% CI 88.9–95.5%) of the patients, respectively (Table 2A). At day 180, the incidence of aGVHD grades II-IV and III-IV was 25.8% (95% CI 22.8–28.9%) vs. 22.6% (95% CI 17.8–27.7%) and 8.5% (95% CI 6.7–10.5) vs. 6.9% (95% CI 4.3–10.4%), respectively (Table 2B, Fig. 1). The 2-year cumulative incidence of all grades and extensive cGVHD was higher in the intermediate compared to the *a*dverse cytogenetics risk group: 40.5% (95% CI 36.8–44.2%) vs. 23.7% (95% CI 18.4–29.4%) and 18.3% (95% CI 15.5–21.3%) vs. 9.3% (95% CI 5.9–13.5%), respectively (Table 2B, Fig. 1).

**Table 2:** A: Outcome—Univariate Analysis; B: Outcome—Univariate Analysis.

A						
Outcomes	*N*	N: Intermediate	N: Adverse	Estimation	Estimation: Intermediate	Estimation: Adverse
**Poly recovery (30** **d)**	1097	820	277	96.2 (94.9–97.2)	96.6 (95.1–97.6)	95.3 (92–97.3)
**Poly recovery (42** **d)**	1097	820	277	97.9 (96.8–98.6)	97.5 (96.2–98.4)	98.9 (96.3–99.7)
**Poly recovery (60** **d)**	1097	820	277	98.2 (97.2–98.8)	97.8 (96.5–98.6)	99.3 (96.5–99.9)
**Platelet recovery (>=20) (60** **d)**	1032	777	255	94.7 (93.2–95.9)	95.3 (93.6–96.6)	92.9 (88.9–95.5)
**Platelet recovery (>=20) (180** **d)**	1032	777	255	95.4 (93.9–96.5)	96 (94.3–97.2)	93.7 (89.8–96.1)
**Platelet recovery (>=50) (60** **d)**	503	375	128	92.2 (89.5–94.3)	92.2 (89–94.6)	92.2 (85.7–95.8)
**Platelet recovery (>=50) (180** **d)**	503	375	128	93.8 (91.3–95.6)	93.3 (90.2–95.5)	95.3 (89.5–97.9)

B					
Estimation	NRM (2 y)	RI (2 y)	LFS (2 y)	OS (2 y)	GRFS (2 y)
**Overall**	19.2 (16.8–21.8)	30.8 (27.9–33.7)	50 (46.8–53.2)	56.4 (53.1–59.5)	38.4 (35.2–41.5)
**Intermediate**	20.5 (17.6–23.5)	24.6 (21.5–27.9)	54.9 (51.1–58.5)	60.6 (56.8–64.2)	41.9 (38.2–45.6)
**Adverse**	15.5 (11.3–20.3)	48.5 (42.1–54.6)	35.9 (29.9–42)	44.4 (38–50.6)	28 (22.3–33.9)

Estimation	aGVH >=II (180 d)	aGVH >=III (180 d)	cGVH (2 y)	extcGVH (2 y)
**Overall**	25 (22.4–27.6)	8.1 (6.5–9.8)	36.3 (33.2–39.4)	16.1 (13.7–18.5)
**Intermediate**	25.8 (22.8–28.9)	8.5 (6.7–10.5)	40.5 (36.8–44.2)	18.3 (15.5–21.3)
**Adverse**	22.6 (17.8–27.7)	6.9 (4.3–10.4)	23.7 (18.4–29.4)	9.3 (5.9–13.5)

*Poly* polymorphonuclears, *d* day.

**Fig. 2:**
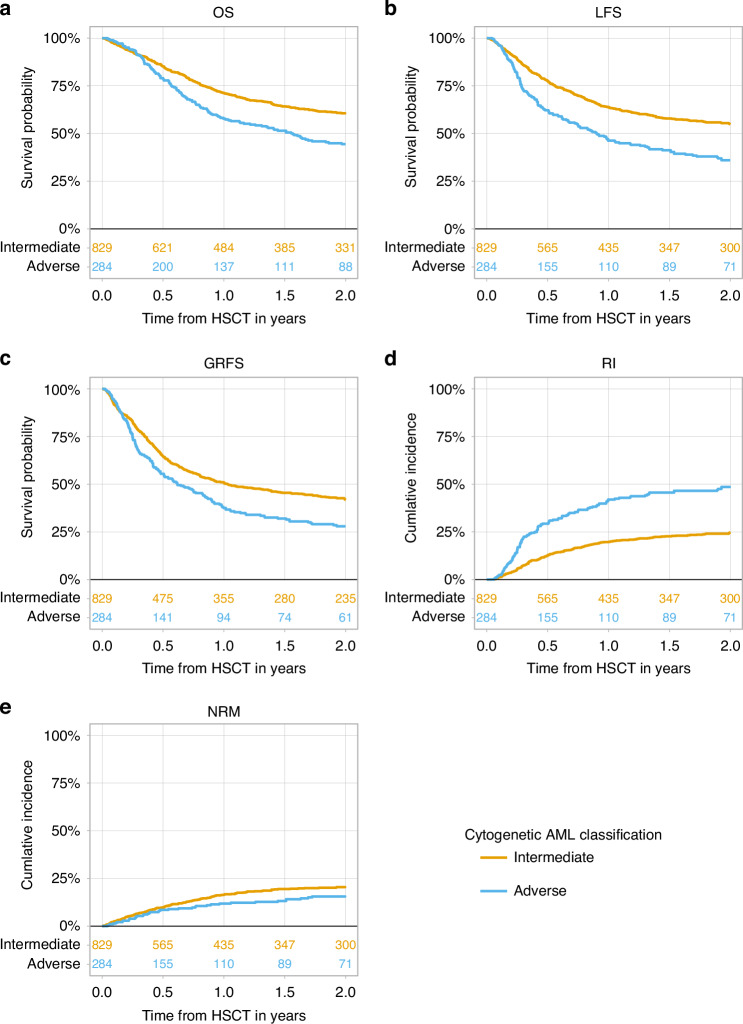
The impact of cytogenetic risk on allogeneic stem cell transplantation outcomes in patients with secondary acute myeloid leukemia. **a** OS-overall survival; **b** LFS- leukemia-free survival; **c** GRFS-graft -versus- host disease- free, relapse- free survival; **d** RI-relapse incidence; **e** NRM-non-relapse mortality.

The 2-year NRM was 20.5% (95% CI 17.6–23.5%) in the intermediate risk group vs. 15.5% (95% CI 11.3–20.3%) in the adverse risk group (Table 2B, Fig. 2). All other HSCT outcomes were inferior in the adverse risk compared to the intermediate risk patients: The 2-year RI was 48.5% (95% CI 42.1–54.6%) vs. 24.6% (95% CI 21.5–27.9%) (Table 2B, Fig. 2). The 2-year LFS, OS, and GRFS were 35.9% (95% CI, 29.9–42%) vs. 54.9% (95% CI, 51.1–58.5%); 44.4% (95% CI, 38–50.6%) vs. 60.6% (95% CI, 56.8–64.2%) and 28.1% (95% CI, 22.3–33.9%) vs. 41.9% (95% CI, 38.2–45.6%), respectively (Table 2B, Fig. 2).

### Multivariate analysis

The incidence of aGVHD grades II-IV and III-IV did not differ between the intermediate risk and adverse risk groups (HR = 0.91, 95% CI: 0.68–1.23; *p* = 0.55 and HR = 0.83 95% CI: 0.5–1.38; *p* = 0.47, respectively). At 2 years, the incidence of all grades and extensive cGVHD was higher in the intermediate compared to the adverse cytogenetics risk group (HR = 0.72, 95% CI: 0.53–0.98; *p* = 0.034 and HR = 0.58, 95% CI: 0.36–0.94; *p* = 0.027, respectively; Table 3). Myeloid engraftment was also higher in the intermediate risk compared to the adverse risk group (HR = 0.85, 95% CI: 0.72–1; *p* = 0.045). Two-year NRM did not differ, HR = 0.78 (95% CI 0.55–1.11, *p* = 0.166). All other HSCT outcomes were inferior in the patients with adverse risk compared to those with intermediate-risk cytogenetics: The HR for 2-year RI was 2.48 (95% CI 1.95–3.15, *p* < 0.001). The HRs for 2-year LFS, OS, and GRFS were 1.62 (95% CI 1.34–1.95, *p* < 0.001), 1.59 (95% CI 1.3–1.93, *p* < 0.001) and 1.38 (95% CI 1.15–1.65, *p* < 0.001), respectively (Table 3). Other significant prognostic factors in the MVA were KPS ≥ 90 which was a prognostic factor for lower NRM, and better LFS, OS and GRFS. Increasing age predicted a higher NRM and a decrease of LFS and OS. CMV seronegativity was associated with a better myeloid engraftment. Unrelated vs. sibling donor was associated with a higher incidence of cGVHD and inferior LFS, GRFS, and myeloid engraftment. Finally, RIC, compared to MAC, was associated with higher RI and lower GRFS (Table 3).

**Table 3:** Outcome—multivariate analysis.

Variable	OS		LFS		GRFS		RI		NRM	
	HR (95%CI)	*P* value	HR (95%CI)	*P* value	HR (95%CI)	*P* value	HR (95%CI)	*P* value	HR (95%CI)	*P* value
**Cytogenetic AML classification: Intermediate VS Adverse**	1.59 (1.31–1.93)	<0.001	1.62 (1.34–1.96)	<0.001	1.38 (1.15–1.65)	<0.001	2.48 (1.95–3.15)	<0.001	0.79 (0.55–1.12)	0.182
**Karnofsky score:** > **= 90 VS** < **90**	1.32 (1.09–1.6)	0.004	1.28 (1.07–1.54)	0.007	1.22 (1.03–1.45)	0.023	1.17 (0.91–1.5)	0.22	1.54 (1.16–2.05)	0.003
**Type of donors: MSD VS UD 10/10**	0.89 (0.73–1.08)	0.249	0.79 (0.65–0.96)	0.014	0.8 (0.67–0.95)	0.013	0.69 (0.54–0.89)	0.004	0.96 (0.71–1.31)	0.796
**Age of the Patient at HCT (per 10 years)**	1.25 (1.12–1.4)	<0.001	1.15 (1.04–1.29)	0.009	1.09 (0.99–1.2)	0.079	1.03 (0.9–1.18)	0.678	1.39 (1.16–1.67)	<0.001
**Female donor to male patient: No VS Yes**	1.05 (0.81–1.35)	0.708	1.04 (0.82–1.32)	0.725	1.13 (0.91–1.4)	0.283	1.15 (0.85–1.56)	0.366	0.89 (0.6–1.31)	0.555
**RIC or MAC regimen: RIC VS MAC**	0.93 (0.76–1.15)	0.516	0.89 (0.73–1.08)	0.242	0.84 (0.7–1.01)	0.07	0.73 (0.55–0.96)	0.023	1.17 (0.86–1.6)	0.317
**CMV Donor to patient: Neg to Neg VS Other**	0.96 (0.77–1.19)	0.691	0.92 (0.75–1.13)	0.438	0.93 (0.77–1.12)	0.454	0.89 (0.67–1.17)	0.394	0.95 (0.69–1.31)	0.742

Variable	aGVH >=II		aGVH >=III		cGVH		extcGVH		Poly recovery	
	HR (95%CI)	*P* value	HR (95%CI)	*P* value	HR (95%CI)	*P* value	HR (95%CI)	*P* value	HR (95%CI)	*P* value
**Cytogenetic AML classification: Intermediate VS Adverse**	0.91 (0.68–1.22)	0.546	0.83 (0.5–1.38)	0.472	0.72 (0.53–0.98)	0.037	0.58 (0.36–0.94)	0.028	0.85 (0.72–0.99)	0.042
**Karnofsky score:** > **= 90 VS** < **90**	0.94 (0.72–1.23)	0.659	1.29 (0.83–1.99)	0.259	1.24 (0.96–1.6)	0.1	1.29 (0.89–1.86)	0.179	1.02 (0.87–1.19)	0.812
**Type of donors: MSD VS UD 10/10**	1.08 (0.82–1.43)	0.59	0.72 (0.46–1.13)	0.152	0.66 (0.51–0.86)	0.002	0.83 (0.57–1.2)	0.321	0.99 (0.85–1.16)	0.93
**Age of the Patient at HCT (per 10 years)**	0.92 (0.8–1.06)	0.232	0.98 (0.76–1.26)	0.887	0.98 (0.86–1.12)	0.766	0.97 (0.79–1.19)	0.756	0.96 (0.88–1.05)	0.354
**Female donor to male patient: No VS Yes**	1.17 (0.84–1.64)	0.348	1 (0.56–1.79)	0.999	0.99 (0.73–1.35)	0.967	1.08 (0.7–1.68)	0.724	0.99 (0.82–1.19)	0.89
**RIC or MAC regimen: RIC VS MAC**	1 (0.76–1.32)	0.994	0.68 (0.41–1.12)	0.127	0.78 (0.59–1.02)	0.074	0.66 (0.44–0.98)	0.042	1.03 (0.87–1.21)	0.764
**CMV Donor to patient: Neg to Neg VS Other**	0.91 (0.68–1.22)	0.521	0.85 (0.52–1.39)	0.518	0.87 (0.66–1.15)	0.332	1.12 (0.75–1.68)	0.584	1.18 (1–1.39)	0.045

*AML* acute myeloid leukemia, *HR* hazard ratio, *IQR* interquartile range, *RI* relapse incidence, *NRM* non-relapse mortality, *LFS* leukemia-free survival, *OS* overall survival, *aGVH* acute graft-versus-host disease, *CGVHD* chronic graft-versus-host disease, *Ext* extensive, *GRFS* GVHD-free and relapse-free survival, *CI* confidence interval, *Poly* polymorphonuclear, *CMV* cytomegalovirus, *RIC* reduced intensity conditioning, *MAC* myeloablative conditioning.

## Cause of death

A total of 480 patients died during the study period—324 in the intermediate-risk patients group and 156 in the adverse-risk group (Table 4). The primary cause of death was the original disease, accounting for 50% and 76% of deaths in the intermediate risk and adverse risk groups, respectively. The second most common cause was HSCT-related complications (infection and GVHD), occurring in 46% and 22% of deaths, respectively (Table 4). Secondary malignancies accounted for 2.8% and 1.3% of the deaths, respectively. Other causes of death were rare (Table 4).

**Table 4:** Cause of death.

Variable	*N*	Intermediate *N* = 829	Adverse *N* = 284
**Main cause of death**	480		
HSCT related		150 (46%)	35 (22%)
Original Disease		163 (50%)	118 (76%)
Secondary malignancy		9 (2.8%)	2 (1.3%)
Other		2 (0.6%)	1 (0.6%)

*HSCT* hematopoietic stem cell transplantation.

## Discussion

In the current study we focused on a large homogenous group of 1119 patients with sAML transplanted from HLA matched sibling or unrelated donor while in CR1. We have demonstrated better outcomes for patients with intermediate-risk cytogenetics defined by ELN2022 compared to outcomes of sAML patients in adverse risk category with better LFS, OS, and GRFS primarly due to lower relapse rate. This finding is expected, as cytogenetic abnormalities detected at the time of diagnosis are well-known independent predictors of the initial response to therapy, remission duration, and OS in AML patients with conventional therapies [26, 27] as well as post-HSCT. However, most prior studies reported the correlation between cytogenetic risk category and post-HSCT survival mainly in de novo AML.

Yanada M and colleagues from Japan assessed the impact of cytogenetic risk on transplantation outcomes of a big cohort of 7812 AML patients demonstrating in MVA the significant effects of cytogenetic risk status on survival irrespective of donor type (related, unrelated, and umbilical cord blood) and even disease status at the time of transplantation (first or second complete remission, and more advanced disease status) [28]. The Center for International Blood and Marrow Transplant Research (CIBMTR), in collaboration with the National Marrow Donor Program (NMDP), assessed transplantation outcomes in 196 patients >60 years of age transplanted in second CR (CR2) (49 of them with sAML). They demonstrated in MVA that cytogenetic risk was the only independent risk factor for OS and relapse, with outcomes being significantly better in patients with intermediate-risk cytogenetics compared to those with unfavorable-risk cytogenetics [29]. The results reported by Tallman et al differ slighty. The authors assessed the impact of the cytogenetic risk group on HSCT outcomes in 261 patients with AML in CR1 and 299 patients in CR2 undergoing matched unrelated HSCT. For patients in first CR, the disease free survival (DFS) and OS at 5 years were similar for the favorable, intermediate, and unfavorable risk groups. In contrast, for patients transplanted in CR2, outcomes were modestly but not significantly better for those with favorable cytogenetics and relapse was somewhat more frequent in patients with unfavorable cytogenetics compared with favorable cytogenetics [30]. Notably, in our cohort focusing on sAML, only 6 patients had favorable cytogenetics risk scores and were therefore not included in the analysis, emphasizing the high risk of the leukemia and the different biology than de novo AML [1–3]. Addressing high risk AML, we at the ALWP conducted a retrospective analysis to determine the clinical outcomes of AML patients undergoing HSCT with respect to specific recurring cytogenetic abnormalities complemented with *FLT3-ITD* status. We analyzed a cohort of 8558 adult AML patients who underwent HSCT from either a matched sibling or a matched unrelated donor demonstrating inferior LFS and patients with adverse cytogenetics and the added prognostic significance of *FLT3-ITD* to baseline cytogenetics in AML patients undergoing HSCT [31]. Regarding FLT3, we subsequently performed a retrospective analysis of 1631 FLT3-ITD AML patients who underwent HSCT demonstrating the influence of cytogenetic risk category in transplanted FLT3-ITD AML patients. On MVA, LFS was significantly lower in patients with intermediate and adverse risk cytogenetics compared to those with favorable risk cytogenetics. OS was significantly lower in patients with adverse risk cytogenetics compared with patients with favorable risk cytogenetics with a trend toward lower OS in patients with intermediate risk cytogenetics compared to those with favorable risk cytogenetics. Finally adverse risk patients and intermediate risk patients experienced higher relapse rates compared with favorable risk patients [18]. Similar findings were observed in the high risk AML subset with pre HSCT positive AML: In MVA, adverse and intermediate/FLT3-ITD3 risk patients were more likely to experience disease relapse compared with favorable risk patients [19]. AML patients with the poorest prognosis are those undergoing HSCT while having active leukemia, either primary refractory or relapsed disease. Poiani M, on behalf of the ALWP, assessed the impact of cytogenetic risk in 2089 patients with refractory or relapsed AML, confirming the prognostic significance of cytogenetics in this very high-risk population. Specifically, compared to the favorable risk group, intermediate and adverse risk patients were associated with worse LFS and OS, as well as a higher incidence of relapse [32]. We subsequently confirmed these findings in AML patients undergoing non-T depleted haploidentical HSCT while having active disease. In MVA, the relapse rate was significantly higher, and LFS and OS significantly lower for patients with adverse risk cytogenetics compared to those with intermediate-risk cytogenetics [17]. As for the sAML subgroup analysis of the MRC 10 trial, which included 1,602 adults with AML (1,797 with de novo AML and 141 with sAML), it demonstrated that cytogenetic risk at diagnosis retained its predictive value in both de novo as well as sAML. Cytogenetic risk was found to be a key determinant of outcomes following HSCT in first CR [33]. Similarly, Armand P and colleagues assessed the prognostic significance of cytogenetic risk in 80 patients with therapy-related AML and reported that cytogenetics was the strongest prognostic factor for relapse and OS. Moreover, after accounting for cytogenetics, patients with therapy-related AML or MDS had an equivalent outcome to those with de novo disease [34]. Similar findings were recently reported in a cohort focusing on MDS, including high-risk patients [35]. Our study aligns with these initial pivotal studies that established the field but now focuses on the impact of cytogenetic risk, as defined by ELN 2022, in a homogenous group of recently transplanted sAML patients, predicting post-transplantation outcomes. Overall, cytogenetic risk predicts transplantation outcomes in high-risk AML, including patients harboring the FLT3+ mutation, those with positive MRD before transplantation as well AML patients active disease at time of transplantation. It is remarkable that, despite significant advances in the field of transplantation [36] and improved outcomes, cytogenetics remains the most important prognostic factor for transplantation success. Furthermore, it is noteworthy that cytogenetic risk is the strongest predicting factor in transplanted sAML patients as within this population many other predicting factors are operating including age, more aggressive biology of the leukemia, lower susceptibility and lower ability to tolerate chemotherapy, and others [1, 6, 7, 9].

The other prognostic factors we observed in the MVA, including age, KPS, CMV serostatus, dose intensity, and donor type, are in agreement with previous publications of allogeneic transplantations, including in sAML [11–15, 37–39]. Being retrospective and registry-based, this transplantation study has several limitations, including the risk of selection bias and the possibility of unavailable data that could not be considered, such as frontline therapies, molecular and MRD data. In summary, our study demonstrated that the cytogenetic risk score defined by ELN2022 predicts HSCT outcomes in a large cohort of sAML patients transplanted between 2010 and 2022. Patients with adverse cytogenetics exhibited significantly higher RI and lower LFS, OS, and GRFS compared to those with intermediate cytogenetics. Notably, the impact of the cytogenetic risk score in sAML is similar to that reported in de novo AML. Hopefully, with the recently approved novel agents for sAML [40], it will be possible to further improve outcomes including in patients with high-risk cytogenetics.

## Supplementary information

Supplementary Material

## Data Availability

AN, ATF, MM and FC had full access to all study data (available upon data-specific request).
