# Supplementary material for: Prognostic significance of cytogenetic risk score in patients with secondary acute myeloid leukemia undergoing allogeneic stem cell transplantation from HLA-matched unrelated donors: a study from the ALWP /EBMT
Source: Bone Marrow Transplant. 2025 May 29;60(9):1218–27. doi: 10.1038/s41409-025-02620-3 (PMC12401728; doi:10.1038/s41409-025-02620-3)
Supplement: Supplementary file 1 — Supplementary Material [file 41409_2025_2620_MOESM1_ESM.docx]

### **Supplemental Table S1: Conditioning regimens**

|  | | | **Cytogenetic classification** | |  |
| --- | --- | --- | --- | --- | --- |
| **Variable** | **N** | **Overall**  N = 1113 | **Intermediate** N = 829 | **Adverse** N = 284 | **p-value** |
| **Conditioning regimen** | 1113 |  |  |  | 0.58 |
| BuFlu-based |  | 502 (45%) | 372 (45%) | 130 (46%) |  |
| TreoFlu-based |  | 188 (17%) | 139 (17%) | 49 (17%) |  |
| FluMel-based |  | 140 (13%) | 111 (13%) | 29 (10%) |  |
| TBI-based |  | 97 (8.7%) | 66 (8.0%) | 31 (11%) |  |
| Other |  | 73 (6.6%) | 53 (6.4%) | 20 (7.0%) |  |
| BuCy-based |  | 61 (5.5%) | 47 (5.7%) | 14 (4.9%) |  |
| TBF-based |  | 52 (4.7%) | 41 (4.9%) | 11 (3.9%) |  |

Abbreviations: TBI-total body irradiation; Mel-melphalan; Bu-busulfan; Flu-

fludarabine; Cy-cytoxan; Treo-treosulfan

**Supplemental Table S2: GVHD prevention**

|  | | | **Cytogenetic AML classification** | |  |
| --- | --- | --- | --- | --- | --- |
| **Variable** | **N** | **Overall**  N = 1113 | **Intermediate** N = 829 | **Adverse** N = 284 | **p-value**^1^ |
| **Associated GVH prevention** | 1113 |  |  |  |  |
| MMF+CSA-based |  | 394 (35%) | 287 (35%) | 107 (38%) |  |
| MTX+CSA-based |  | 375 (34%) | 271 (33%) | 104 (37%) |  |
| CSA-based |  | 171 (15%) | 134 (16%) | 37 (13%) |  |
| MMF+TACRO-based |  | 72 (6.5%) | 61 (7.4%) | 11 (3.9%) |  |
| Other |  | 21 (1.9%) | 17 (2.1%) | 4 (1.4%) |  |
| MTX+TACRO-based |  | 19 (1.7%) | 13 (1.6%) | 6 (2.1%) |  |
| TACRO-based |  | 14 (1.3%) | 9 (1.1%) | 5 (1.8%) |  |
| TACRO+SIRO-based |  | 14 (1.3%) | 11 (1.3%) | 3 (1.1%) |  |
| MMF-based |  | 11 (1.0%) | 8 (1.0%) | 3 (1.1%) |  |
| MMF+SIRO-based |  | 11 (1.0%) | 9 (1.1%) | 2 (0.7%) |  |
| MMF+CSA+TACRO-based |  | 5 (0.4%) | 4 (0.5%) | 1 (0.4%) |  |
| MTX+CSA+TACRO-based |  | 5 (0.4%) | 4 (0.5%) | 1 (0.4%) |  |
| MTX-based |  | 1 (<0.1%) | 1 (0.1%) | 0 (0%) |  |

Abbreviations: GVHD-graft versus host disease; CSA- cyclosporine A; MTX-

Methotrexate; MMF- mycophenolate mofetil; SIRO- sirolimus; TACRO- tacrolimus.

**Contributing Centers**

Hannover Medical School, Hannover, Germany; University Hospital Eppendorf, Hamburg, Germany; University of Muenster, Muenster, Germany; University Hospital Gasthuisberg, Leuven, Belgium; Saint-Louis Hospital, BMT Unit, Paris, France; Medizinische Klinik m. S. Hämatologie, Onkologie und Tumorimmunologie, Berlin, Germany; University Hospital | Essen, Essen, Germany; Dél-pesti Centrumkórház, Budapest, Hungary; Oslo University Hospital, Rikshospitalet, Oslo, Norway; University Hospital Frankfurt - Goethe University, Frankfurt Main, Germany; Hopital Saint Antoine, Paris, France; Programme de Transplantation & Therapie Cellulaire, Marseille, France; University Medical Centre Utrecht, Utrecht, Netherlands; Universite Paris IV, Hopital la Pitié-Salpêtrière, Paris, France; CHU Bordeaux, Hopital Haut-Leveque, Pessac, France; Centre Hospitalier Lyon Sud, Lyon, France; Azienda Ospedaliero Universitaria di Udine, Udine, Italy; Universitaetsklinikum Dresden, Dresden, Germany; Demiroglu Bilim University Istanbul Florence Nightingale Hospital, Istanbul, Turkey; Cliniques Universitaires St. Luc, Brussels, Belgium; Erasmus MC Cancer Institute, Rotterdam, Netherlands; Hope Directorate, Dublin, Ireland; CHU Grenoble Alpes - Université Grenoble Alpes, Grenoble, France; Medical Clinic and Policinic 1, Leipzig, Germany; University Medical Center Groningen (UMCG), Groningen, Netherlands; Rigshospitalet, Herlev, Denmark; Hopital Jean Minjoz, Besancon, France; HUCH Comprehensive Cancer Center, Helsinki, Finland; Gustave Roussy Cancer Campus, Villejuif, France; CHU Lapeyronie, Montpellier, France; Robert_Bosch_Krankenhaus, Stuttgart, Germany; Universitaet Tuebingen, Tuebingen, Germany; CHU de Lille, Lille, France; University Hospitals Bristol and Weston NHSFT, Bristol, United Kingdom; Universitaetsklinikum Jena, Jena, Germany; Klinikum Rechts der Isar, Munich, Germany; C.H.R.U de Brest, Brest, France; Ospedale San Raffaele s.r.l., Milano, Italy; Derriford Hospital Plymouth, Plymouth, United Kingdom; USD Trapianti di Midollo, Adulti, Brescia, Italy; Leiden University Hospital, Leiden, Netherlands; CHU Nantes, Nantes, France; CHU ESTAING, Clermont, France; RVI Newcastle, Newcastle, United Kingdom; University Hospital Aachen, Aachen, Germany; Universitaetsklinikum Goettingen, Goettingen, Germany; Addenbrookes Hospital Cambridge, Cambridge, United Kingdom; Secretary and Italian National BMT Registry - GITMO, Bergamo, Italy; Centre Hospitalier Universitaire de Rennes, Rennes, France; ICANS - Institut de cancérologie Strasbourg Europe, Strasbourg, France; Kings College Hospital London, London, United Kingdom; University of Freiburg, Freiburg, Germany; Klinikum Augsburg, Augsburg, Germany; University Hospital | Basel, Basel, Switzerland; Turku University Hospital, Turku, Finland; Hospital U. Marqués de Valdecilla, Santander, Spain; Birmingham Centre for Cellular Therapy and Transplant (BCCTT), Stoke, United Kingdom; University of Heidelberg, Heidelberg, Germany; CHU - Institut Universitaire du Cancer Toulouse, Toulouse, France; Charles University Hospital, Pilsen, Czech Republic; Christie Hospital Manchester, Manchester, United Kingdom; CHRU Limoges, Limoges, France; Klinik fuer Innere Medzin III, Ulm, Germany; Institut Jules Bordet, Brussels, Belgium; S.S.C.V.D Trapianto di Cellule Staminali, Torino, Italy; University Medical Center Schleswig-Holstein, Campus Kiel, Kiel, Germany; Hospital Santa Creu i Sant Pau, Barcelona, Spain; University Hospital | Uppsala, Uppsala, Sweden; University Medical Center Schleswig-Holstein, Luebeck, Germany; A.Z. Sint-Jan, Gent, Belgium; VU University Medical Center, Amsterdam, Netherlands; Aarhus University Hospital | Denmark, Aarhus, Denmark; Sheffield Royal Hallamshire, Sheffield, United Kingdom; Klinikum Chemnitz gGmbH, Chemnitz, Germany; Karolinska University Hospital, Stockholm, Sweden; Royal Marsden Hospital, London, United Kingdom; Hopital La Miletrie, Poitiers, France; Skanes University Hospital, Lund, Sweden; Asst Grande Ospedale Metropolitano Niguarda, Milano, Italy; Cardiff University Hospital of Wales & Swansea, Cardiff, United Kingdom; Rambam Medical Center, Haifa, Israel; Klinikum Grosshadern, Munich, Germany; CHU Nice - Hôpital de l’Archet I, Nice, France; University Hospital Maastricht, Maastricht, Netherlands; Southampton General Hospital, Southampton, United Kingdom; Nottingham City Hospital, Nottingham, United Kingdom; University of Liege, Liege, Belgium; University of Saarland, Homburg, Germany; Centre Henri Becquerel, Rouen, France; Universitaet Bonn, Bonn, Germany; Hospital Clinic, Barcelona, Spain; Ospedale Civile, Pescara, Italy; Institut de Cancerologie Lucien Neuwirth, Saint Etienne, France; CHU Caen, Caen, France; Research Committee - University of Patras, Patras, Greece; Hospital San Maurizio, Bolzano, Italy; Universitaetsklinium Magdeburg, Magdeburg, Germany; University of Cologne, Cologne, Germany; Department of Hematology and Transplantology of Lower Silesian Center of  Oncology, Wroclaw, Poland; George Papanicolaou General Hospital, Thessaloniki, Greece; Hospital Universitario de Navarra, Pamplona, Spain; University Hospital La Fe, Valencia, Spain; Hospital Clínico, Salamanca, Spain; Ghent University Hospital, Gent, Belgium; Tartu University Hospital, Tartu, Estonia; University Medical Center Mainz, Mainz, Germany; S. Bortolo Hospital, Vicenza, Italy; ZSIS Universitaetsklinikum Knappschaftskrankenhaus Bochum GmbH, Bochum, Germany; Nijmegen Medical Centre, Nijmegen, Netherlands; Bologna University, S.Orsola-Malpighi Hospital, Bologna, Italy; Glasgow Royal Infirmary, London, United Kingdom; Birmingham Centre for Cellular Therapy and Transplant (BCCTT), Birmingham, United Kingdom; Ospedale Infantile Regina Margherita, Torino, Italy; Istituto Clinico Humanitas, Milano, Italy; American University of Beirut Medical Center, Beirut, Lebanon; Clatterbridge Cancer Centre Liverpool, Liverpool, United Kingdom; Hospital Vall d`Hebron, Barcelona, Spain; University Hospital Brno, Brno, Czech Republic; University Hospital, Bratislava, Slovak Republic; Vilnius University Hospital Santaros Klinikos, Vilnius, Lithuania; Hôpital  D'Instruction des Armées (HIA) PERCY, Clamart, France; CHRU Nancy, Vandoeuvre les Nancy, France; RM Gorbacheva Research Institute, Pavlov University, Petersburg, Russian Federation; Chaim Sheba Medical Center, Tel-Hashomer, Israel; ¨Tor Vergata¨ University of Rome, Rome, Italy; Institut Catalá d`Oncologia, Hospital Duran i Reynals, Barcelona, Spain; University Hospital Erlangen, Erlangen, Germany; H SS. Antonio e Biagio, Alessandria, Italy; Medicana International Hospital Istanbul, Istanbul, Turkey; University of Amiens: CHU Amiens, Amiens, France; Antwerp University Hospital (UZA), Antwerp E, Belgium; Mazzoni Hospital, Ascoli Piceno, Italy; Universitaetsmedizin Mannheim, Mannheim, Germany; University Hospital, Zürich, Switzerland; Hospital Sirio-Libanes, Sao Paulo, Brazil; University College London Hospital, London, United Kingdom; Hospital de la Princesa, Madrid, Spain; Hôpital Henri Mondor, Creteil, France; Hadassah University Hospital, Jerusalem, Israel; Sahlgrenska University Hospital, Goeteborg, Sweden; University Hospital Center Rebro, Zagreb, Croatia; U.O.D Trapianti di midollo osseo, Rozzano, Italy; Ospedale Dell'Angelo, Venezia, Italy; Ospedale San Gerardo, Monza, Italy; Grande Ospedale Metropolitano Bianchi Melacrino Morelli - Centro Unico Trapianti A. Neri, Reggio Calabria, Italy; Manchester Royal Infirmary, Manchester, United Kingdom; Az. Ospedaliera S. Croce e Carle, Cuneo, Italy; Hospital Universitario Central de Asturias, Oviedo, Spain; CHRU, Angers, France; Institute of Hematology and Blood Transfusion, Prague, Czech Republic; Leicester Royal Infirmary, Leicester, United Kingdom; University Regensburg, Regensburg, Germany; Azienda Ospedali Riuniti di Ancona, Ancona, Italy; Hospital Gregorio Marañón, Madrid, Spain; St. Franziskus Hospital, Munich, Germany; San Matteo Pavia Transplant Programme, Pavia, Italy; Institutul Regional de Oncologie, Iași, Romania; Asklepios Klinik St. Georg, Hamburg, Germany; University Hospital Ostrava, Ostrava, Czech Republic; Hopital Necker Adults, Paris, France; Tel Aviv Sourasky Medical Center, Tel Aviv, Israel; Gazi University Faculty of Medicine, Ankara, Turkey; Imperial College Hammersmith London, London, United Kingdom; Medizinische Universitaet Wien, Vienna, Austria; Univ. La Sapienza, Rome, Italy; Univ. of Parma, Parma, Italy; St James University Hospital Leeds, Leeds, United Kingdom; Département d'Oncologie, Service d'Hématologie, Geneva, Switzerland; Belfast City Hospital, Belfast, United Kingdom; Hospital Clínico de Valencia, Valencia, Spain; Klinikum Karlsruhe gGmbH, Karlsruhe, Germany; Inst. Português de Oncologia do Porto, Porto, Portugal; Inst. Portugues Oncologia, Lisboa, Portugal; Universita Cattolica S. Cuore, Rome, Italy; LKH - University Hospital Graz, Graz, Austria; Fundación Jiménez Díaz, Madrid, Spain; Martin-Luther-Universitaet Halle-Wittenberg, Halle, Germany; Heinrich Heine Universitaet, Duesseldorf, Germany; King Faisal Specialist Hospital & Research Centre, Riyadh, Saudi Arabia; Beilinson Hospital, Petach-Tikva, Israel; Ankara Bayindir Hospital, Ankara, Turkey; Department of Bone Marrow Transplantation and Oncohematology, Gliwice, Poland; Singapore General Hospital, Singapore, Singapore; King Abdul - Aziz Medical City, Riyadh, Saudi Arabia; Klinik fuer Innere Medizin C, Greifswald, Germany; Hospital de Gran Canaria Dr Negrin, Las Palmas, Spain; Hospital Univ. Virgen de las Nieves, Granada, Spain; Clinic of Hematology, Belgrade, Serbia; LKH - University Hospital Graz, Graz, Austria; Elisabethinen-Hospital, Linz, Austria; ICO-Hospital Universitari Germans Trias i Pujol, Badalona, Spain; Ankara University Faculty of Medicine, Ankara, Turkey; Policlinico G.B. Rossi, Verona, Italy; Klinikum Nuernberg, Wuerzburg, Germany; Clinical Hospital Merkur, Zagreb, Croatia; Philipps Universitaet Marburg, Marburg, Germany; AZ Delta, Roeselare, Belgium; U.O. Ematologia con Trapianto, Bari, Italy; Arcispedale S. Maria Nuova, Reggio E, Italy; Institute of Hematology and Transfusion Medicine, Warsaw, Poland; Clinica Puerta de Hierro, Madrid, Spain; Charles University Hospital, Hradec, Czech Republic; Umea University Hospital, Umeå, Sweden; Clínica Universitaria de Navarra, Pamplona, Spain; Klinikum Oldenburg, Oldenburg, Germany; St. Savvas Oncology Hospital, Athens, Greece; St. Bartholomew`s Hospital London, London, United Kingdom; Azienda Ospedaliero Universitaria Pisana, Pisa, Italy; University Clinical Centre in Gdansk, Gdansk, Poland; King Faisal Specialist Hospital and Research Center, Jeddah, Saudi Arabia; First Affiliated Hospital of Soochow University, Suzhou, China; San Matteo Pavia Transplant Programme, Pavia, Italy
